# The Classical Apoptotic Adaptor FADD Regulates Glycolytic Capacity in Acute Lymphoblastic Leukemia

**DOI:** 10.7150/ijbs.68016

**Published:** 2022-05-01

**Authors:** Wenzhao Zhou, Yueyang Lai, Jianhui Zhu, Xuebo Xu, Wenliang Yu, Zengzheng Du, Leyang Wu, Xuerui Zhang, Zichun Hua

**Affiliations:** 1 The State Key Laboratory of Pharmaceutical Biotechnology, School of Life Sciences, Nanjing University, Nanjing, China.; 2 School of Biopharmacy, China Pharmaceutical University, Nanjing, China.; 3 Changzhou High-Tech Research Institute of Nanjing University and Jiangsu Target Pharma Laboratories Inc., Changzhou, China.; 4 Shenzhen Research Institute of Nanjing University, Shenzhen, China.

**Keywords:** FADD, ALL, glycolysis, aerobic respiration, single nucleotide polymorphism, apoptosis

## Abstract

The Fas-associated death domain (FADD) has long been regarded as a crucial adaptor protein in the extrinsic apoptotic pathway. Despite the non-apoptotic function of FADD is gradually being discovered and confirmed, its corresponding physiological and pathological significance is still unclear. Based on the database of GWAS catalog and GTEx Portal, 17 SNPs associated with leukemia susceptibility were found to be linked to FADD expression. We then investigated a regulatory role of FADD in T-acute lymphoblastic leukemia (T-ALL) using Jurkat cells as a model. Jurkat cells stably depleted of FADD (FADD^-/-^ Jurkat) expression exhibited dampened proliferation, hypersensitivity to Etoposide-induced intrinsic apoptosis whereas near total resistance to TRAIL-induced extrinsic apoptosis. Comparison between wild type and FADD^-/-^ Jurkat cells using iTRAQ-based proteomics revealed considerably altered expression spectrum of genes, and led us to focus on metabolic pathways. Investigation of glycolytic and mitochondrial pathways and relevant enzymes revealed that FADD knockout triggered a metabolic shift from glycolysis to mitochondrial respiration in Jurkat cells. Re-expression of FADD in FADD^-/-^ Jurkat cells partially rescued glycolytic capacity. FADD loss triggers global metabolic reprogramming in Jurkat cells and therefore remains as a potential druggable target for ALL treatment.

## 1. Introduction

Fas-associated death domain (FADD) is a pivotal factor in relaying apoptotic signals. Several membrane-bound death receptors such as FasL, TNFR1, and TRAILR routinely recruit intracellular FADD upon binding extracellular ligands. Association of FADD with death receptors further attracts downstream Caspase to form the famous death-inducing signaling complex (DISC) to execute apoptosis 1. Although discovered as an adaptor protein in extrinsic apoptotic pathway, FADD has been reported to engage in multiple non-apoptotic processes including lymphocyte proliferation and activation, cell cycle progression, and embryonic development 2-4. T-lymphocytes bearing loss of FADD displayed retarded proliferation and cell cycle. Our research group has also identified a regulatory role of FADD in metabolism 5, 6. Overwhelming evidence points to versatile non-apoptotic functions of FADD and many more remain to be discovered.

Cell metabolism is indispensable and orchestrated by thousands of enzymes which catalyze conversion of nutrients into absorbable small molecules essential for all cellular processes. Mitochondrion is the center for metabolism at which oxidative phosphorylation, fatty acid oxidation, and tricarboxylic acid (TCA) cycle take place 7. Built as one of the vital organelles for cell survival, mitochondria of a cell determine its fate and therefore are effective targets for fine-tuning cell processes such as growth and death^8^. Cytochrome c is located within the compartment encompassed by the inner and outer membranes of mitochondrion. It transfers electrons in the respiratory chain for generation of ATP. It is also released from mitochondria as a cell is committed to apoptosis 9. The Bcl-2 family, which consists of both pro-apoptotic (e.g. Bak) and anti- apoptotic (e.g. Bcl-2) proteins, has been reported to modulate the permeability of mitochondrial membrane which directly relates to the release of cytochrome c 10. The Voltage Dependent Anion selective Channels (VDACs), are pore-forming proteins and function by adjusting the pore size of mitochondrial membrane via forming either monomers (smaller size) or polymers (larger size) as a response to reactive oxygen species (ROS) signaling. VDACs can regulate the integrity of mitochondrial membrane by adjusting pore sizes and therefore cytochrome c release which marks commitment to apoptosis^11^. Other reports have revealed c-RAF's interaction with VDACs, which rendered c-RAF a potential regulator of mitochondrial function 12.

Cancer has long been recognized as a disorder of uncontrolled growth 13. Although the Warburg effect was proposed back in 1931 and was a Nobel prize-winning discovery, it was not until a decade ago did scientists begin to value the benefits brought by glycolysis to cancer cells 14. Different types of cancer share few mutations yet they all rely heavily on glycolysis as an important energy source 15. They are reprogrammed towards glycolysis. Regardless of oxygen availability, cancer cells uptake large amount of glucose for glycolysis occurring in the cytoplasm, producing lactate as a major byproduct. Accumulation of lactate directly contributes to acidic tumor microenvironment (TME) which in turn promotes expression of certain oncogenes, e.g. EGFR and VEGF, favorable for cancer progression, metastasis and invasion 16. Furthermore, excessive ROS hinders cancer growth. Systemic metabolic shift towards glycolysis in cancer cells restrains ROS production which is a major byproduct of oxidative phosphorylation 17. Expression of several key enzymes of glycolysis are increased in cancer. HK2, which catalyzes the first and rate-limiting step in glycolysis, phosphorylates glucose to produce glucose-6-phosphate. Although glycolysis takes place in the cytoplasm, HK2 tend to form complex with mitochondrial VDAC to gain access to mitochondrially produced ATP which is a phosphate donor 18. VDAC-HK2 complex has been studied extensively and their expression are changed concurrently 19.

In this work we revealed a connection between FADD and metabolism in ALL using the Jurkat cell line as a model. We performed a bioinformatic analysis in an attempt to discover SNP loci associated with both leukemia susceptibility and FADD expression using the database of GWAS catalog and GTEx Portal. We then constructed a Jurkat cell line permanently deprived of FADD using CRISPR. Unexpectedly, although loss of FADD did confer resistance to extrinsic apoptosis induced by TRAIL, FADD^-/-^ Jurkat cells exhibited dampened proliferation and increased degree of intrinsic apoptosis induced by Etoposide. Comparative proteomics showed considerably altered expression spectrum in Jurkat cells upon FADD loss. Further analysis of proteomic data by Metacore and customized scripts revealed mitochondrial and glycolytic pathways were among many affected by FADD loss. Investigation of metabolic enzymes revealed diminished expression of key enzymes in glycolysis. Interestingly, expression of key enzymes in mitochondrial respiration were increased. Seahorse analysis revealed an increase in capacity for mitochondrial respiration concomitant with a decrease in glycolytic in FADD^-/-^ Jurkat cells. Further experiments revealed an increase in the number of mitochondria in FADD^-/-^ Jurkat cells. On the other hand, we examined intrinsic apoptosis-related proteins as FADD^-/-^ Jurkat cells exhibited hypersensitivity to Etoposide. Proteomic data along with immunoblot results revealed elevated VDAC and Bak expression whereas decreased c-Raf and Bcl-2 expression upon FADD loss, along with an increase in ROS production. Lastly, we perform a rescue experiment by re-expressing FADD and phosphorylated FADD (pFADD) mimic FADD-D in FADD^-/-^ Jurkat cells. Re-expression of FADD in FADD^-/-^ Jurkat cells partially restored oncogenic ability and glycolytic capacity while FADD-D failed to achieve any restoration. Our data suggest a regulatory role in glucose utilization and metabolism in ALL and therefore potentiates FADD as a druggable target for ALL treatment.

## 2. Methods and Materials

### 2.1 Data collection for SNP analysis

A search was performed on the GWAS Catalog online database (https://www.ebi.ac.uk/gwas/) as of Nov. 22, 2020 using key words Acute Lymphoblastic Leukemia and p-value cutoff of 1 x 10^-5^. Search results from the GWAS Catalog were further processed using the GTEx eQTL Calculator to seek their connections to FADD in whole blood samples (https://gtexportal.org/home/testyourown) using a p-value cutoff of 0.05.

### 2.2 Cell culture

The Jurkat cell line (T-cell leukemia, ATCC® TIB-152™) was purchased from American Type Culture Collection and cultured in RPMI1640 medium supplemented with 10% (v/v) fetal bovine serum (FBS, Gibco) and 1% (v/v) penicillin-streptomycin (Gibco).

### 2.3 FADD knockout in Jurkat cells using CRISPR/Cas9

FADD knockout in Jurkat T cells was achieved using the CRISPR/Cas9 system as described previously 20. Two single guide RNAs (sgRNAs) both targeting the FADD gene were designed using an online CRISPR design platform (https://zlab.bio/guide-design-resources). Sequences of the two sgRNAs are 5'-TTCCTATGCCTCGGGCGCGT-3' and 5'- GCGTCGACGACTTCGAGGCG-3'. Two sgRNA sequences were respectively cloned into separate pLentiCRISPRv2 plasmids. HEK293T cells were co-transfected with constructed pLentiCRISPRv2 plasmids and lentiviral envelope plasmids (PL3, PL4, and PL5). Viruses were extracted three days following transfection by ultracentrifugation (72000 g for 2h). Jurkat cells were then treated by viruses and screened by puromycin (2 μg/ml) and single cell clones were isolated by serial dilutions, denoted as FADD^-/-^ Jurkat cells. FADD knockout in two FADD^-/-^ Jurkat cell lines was identified by Western blot. For construction of FADD-D Jurkat cells, FADD^-/-^ Jurkat cells were transfected with lentivirus expressing FADD-S194D.

### 2.4 Cell proliferation assay

Proliferation of Jurkat cells was monitored using the CCK-8 kit (Beyotime, Nantong, China) according to the manufacturer's instructions. Briefly, 5×10^3^ cells were seeded into each well of 96-well plates. Samples were measured at 48 h post-seeding in triplicate at 450 nm by the Tecan Infinite 200 PRO plate reader (Tecan Group Ltd., Männedorf, Switzerland).

### 2.5 Apoptosis treatment

Jurkat cells, either wild type or FADD^-/-^, were treated with apoptosis inducers, Etoposide (100 μM) or TRAIL (4 μg/mL).

### 2.6 Annexin V-Propidium Iodide (PI) double staining using flow cytometry

Jurkat cells were treated by Annexin V-FITC in Ca^2+^-containing binding buffer at room temperature for 30 min. PI was added 5 min before flow cytometric measurement. Degree of apoptosis was determined by Annexin V (green) and PI (red) fluorescence.

### 2.7 Determination of ROS Generation

The ROS detection kit (KeyGen Biotech., Nanjing, China) was used to measure ROS production in both wild type and FADD^-/-^ Jurkat cells subjected to different treatment. Briefly, 2',7' - dichlorofluorescein diacetate (DCFH-DA) was diluted to a concentration of 10 mM using RPMI-1640 medium. 10^6^ cells were collected and resuspended in medium containing DCFH-DA, followed by incubation at 37 ℃ for 20 min. Green fluorescence of cells was detected by flow cytometry.

### 2.8 Western blot analysis

Cells were collected and washed twice with ice-cold PBS, followed by lysis using RIPA buffer (protein inhibitor included) for 40 min. Lysed cells were precipitated by centrifugation (12,000 rpm for 10 min at 4 °C) and cell extracts in the supernatant were harvested. Proteins in the supernatant were separated using 12% SDS-PAGE and transferred to PVDF membranes (Millipore, Bedford, MA, USA). Membranes were then blocked in non-fat 5% milk at room temperature for 1 h and blotted with appropriate primary antibodies at 4 °C overnight. Following three washes in PBS (10 mM, pH 7.4) containing 0.1% Tween 20, membranes were blotted with secondary antibodies of either mouse or rabbit origin for 1 h at room temperature. After three washes in PBS with 0.1% Tween 20, the blots were visualized using ECL detection reagents. Bands were quantified by the ImageJ software.

### 2.9 Protein sample preparations for comparative proteomics

Jurkat cells were collected and washed three times with ice-cold PBS. Cell precipitates were then treated with 200 mL TEAB (0.5 mol/L triethylammonium bicarbonate) dissolution buffer and sonicated for 20 min, followed by centrifugation at 12,000 r/min for 15 min. The supernatant was collected and mixed with 4-fold volume of cold acetone containing 10 mmol/L DTT and the mixture was let stand for 2 h. After centrifugation at 12,000 r/min for 15 min at 4°C, the pellets were collected and resuspended by 800 mL cold acetone at 56 °C to break disulfide bonds. Pellets were again precipitated by centrifugation at 12,000 r/min for 15 min at 4 °C, and collected and again dissolved by 100 mL TEAB dissolution buffer. Protein concentration was determined by the Bradford protein method. An aliquot of the solution (100 mg of total proteins) was adjusted to a volume of 100 mL using TEAM dissolution buffer and then diluted with 500 mL 50 mmol/L NH_4_HCO_3_. 2 mg trypsin was added and then incubated overnight at 37 °C. Following protein digestion, equal volume of 0.1% formic acid (600 mL) was added for acidification. Peptides were purified on a Strata-XC18 pillar which was pre-treated sequentially with methanol, 1 mL 0.1% formic acid (three times), 0.1% formic acid + 5% acetonitrile (twice), and 1 mL 0.1% formic acid + 80% acetonitrile. Peptides were then dried by vacuum centrifugation and redissolved in 20 mL 0.5 mol/L TEAB for peptides labeling.

Peptides were then labeled with iTRAQ Reagent-8 plex Multiplex Kit (AB Sciex U.K. Limited) according to the manufacturer's instructions. Samples and labeled marker were as follows: Samples from Jurkat cells were labeled with iTRAQ tag 113 、samples from FADD^-/-^ Jurkat cells were labeled with iTRAQ tag 114、samples from apoptotic Jurkat cells were labeled with iTRAQ tag 119 and samples from apoptotic FADD^-/-^ Jurkat cells were labeled with iTRAQ tag 121 (J113, F114, AJ119, AF121). All of the labeled samples were mixed with an equal amount. The labeled samples were fractionated using high-performance liquid chromatography (HPLC) system (Thermo DINOEX Ultimate 3000 BioRS) using a Durashell C18 (5 μm, 100 A, 4.6 × 250 mm).

### 2.10 LC-MS/MS

Proteomic data was acquired on a Triple TOF 5600 System (AB SCIEX, Concord, ON). Samples were chromatographically separated using a 90 min gradient from 2%-30% (mobile phase A 0.1% (v/v) formic acid, 5% (v/v) acetonitrile; mobile phase B 0.1% (v/v) formic acid, 95% (v/v) acetonitrile) after direct injection onto a 20 μm PicoFrit emitter (New Objective) packed to 12 cm with Magic C18 AQ 3 μm 120 A stationary phase. MS1 spectra were collected in the range 350-1,500 m/z for 250 ms. The 20 most intense precursors with charge state 2-5 were selected for fragmentation, and MS2 spectra were collected in the range 50-2,000 m/z for 100 ms; precursor ions were excluded from reselection for 15 s.

### 2.11 Data analysis

Protein identification and quantification was achieved using the ProteinPilot Software 5.0 (AB SCIEX) which employs the Paragon™ database search algorithm (5.0.0.0.4767) and non-linear fitting method to determine the integrated false discovery rate (FDR) for peptide identification and quantification 21. An automatic decoy database search strategy 22 was used to estimate FDR using the PSPEP (Proteomics System Performance Evaluation Pipeline Software) algorithm. Proteins of at least one unique peptide and an FDR value greater than 1.3 were selected for further analysis. Proteomic data were interpreted according to fold change in expression. The cut-off for high abundance (> 1.2-fold over normal, P < 0.05) and low abundance (< 0.8-fold over normal, P < 0.05) proteins were selected to identify differentially expressed proteins based on biological replicate method.

### 2.12 Mitochondrial DNA quantification

Mitochondrial DNA (mtDNA) was quantified as previously described 23. Briefly, total genomic DNA of cells was extracted using the Genomic DNA mini preparation Kit (Beyotime, Beijing, China). Mitochondrial DNA level was determined based on the relative level of cytochrome oxidase 1 compared to β-globin.

### 2.13 Determination of glucose consumption, and ATP, acetate, pyruvate and production

Cells were cultured in FBS-free medium overnight prior to measurement. Glucose consumption, lactate, ATP, pyruvate production assay kits were all purchased from Solarbio (Beijing, China) and used according to manufacturer's instructions. All experiments were performed in triplicates.

### 2.14 Measurements of oxygen consumption rates and extracellular acidification rates

Mitochondrial oxygen consumption rates (OCR) and extracellular acidification rates (ECAR) were assessed using a Seahorse XF24 Extracellular Flux Analyzer (Seahorse Bioscience). Wild type and FADD^-/-^ Jurkat cells were seeded at a density of 20,000 cells in XF24 cell culture microplate. After 8 h incubation, cells were continuously incubated for 12 h in hypoxia. Before the assay, the culture medium was changed to XF base media as recommended by Seahorse Bioscience. The ATP synthase inhibitor Oligomycin A (2 μM), the ATP synthesis uncoupler carbonyl cyanide-4-trifluoromethoxyphenylhydrazone FCCP (1.5 μM), the complex I inhibitor rotenone (2 μM), and complex III inhibitor antimycin A (2 μM) were used to determine OCR parameters. OCR is shown in pmol/min. All experiments were performed at least three times.

### 2.15 Bioinformatics analysis

Protein expression profiles were analyzed by pathway analysis, enrichment analysis of MetaCoreTM version 5.4 (GeneGo, St. Joseph, MI). For enrichment analysis, gene IDs of the uploaded files were matched with gene IDs in GeneGo ontologies in MetaCoreTM which includes GeneGo Pathway Maps, GeneGo Process Networks, GeneGo Metabolic Networks and GO Processes. For network analysis, shortest paths algorithms were used.

Protein expression heat maps, volcano plots, bubble charts, polar coordinate plots are constructed either by R or python. Details are in the legends of respective figures.

### 2.16 Statistical analysis

Data in this study are presented as mean ± SD. Student's t-test was used for statistical analysis. All statistical analyses were conducted using GraphPad Prism 6. P-value less than 0.05, 0.01, 0.001, and 0.0001 is indicated by *, **, ***, and ****, respectively.

## Results

### 3.1 Single Nucleotide Polymorphism (SNP) analysis connects FADD to ALL

**Figure 1:**
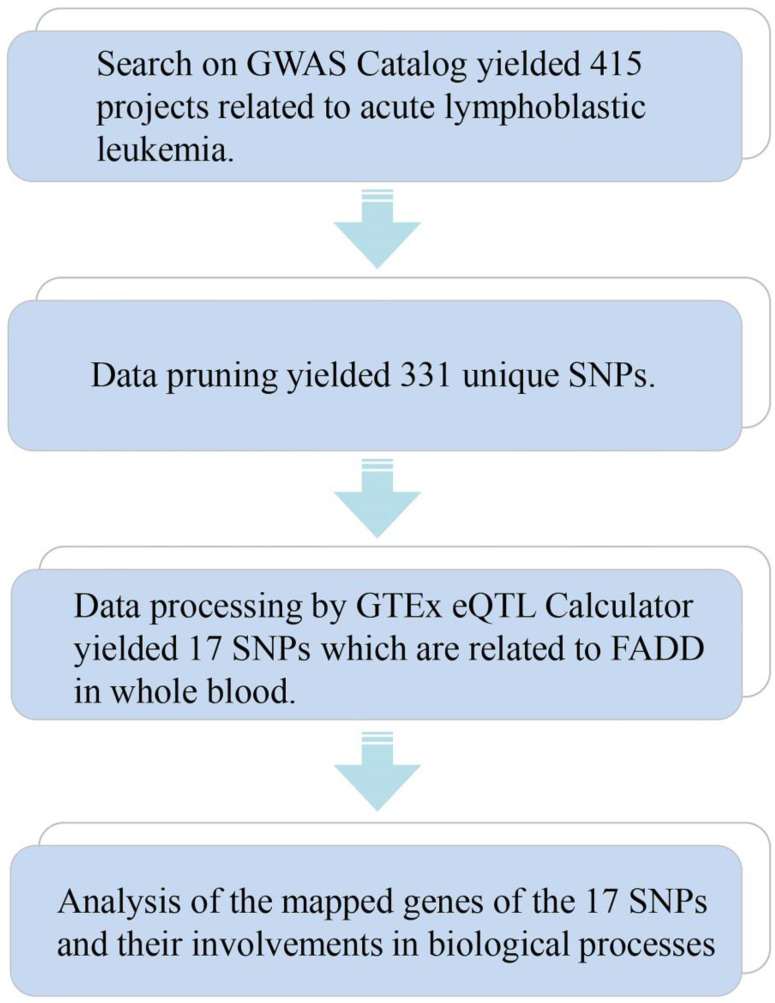
SNP analysis connects FADD to ALL. A flow chart of SNP analysis. Details of the analytic process is shown in the Methods and Materials section.

We employed means of bioinformatics to seek connections between FADD and Acute Lymphoid Leukemia (ALL). The bioinformatics method is demonstrated in Figure 1. 331 unique SNPs related to ALL were found (415 projects, Data as of 2020.11.22) on the GWAS catalog database. SNPs were further processed using the GTEx eQTL Calculator to seek their connections to FADD in whole blood samples, and 17 SNPs were discovered to be closely related to FADD in whole blood (*P* < 0.05) (Table 1). Further analysis of mapped genes of these 17 SNPs revealed the following outcome: 4 SNPs are involved in metabolic process, and 2 SNPs are involved in intrinsic apoptosis. More details regarding these 17 SNPs are listed in Supplementary Table 1. Although further bioinformatics analysis was not pursued, our results showed a link between FADD and ALL.

Under physiological and pathological conditions, FADD knockout is far less likely to occur compared to hyper- or hypo-phosphorylation of FADD. Previous studies of our group showed that FADD knockout is phenotypically similar to hyper-FADD phosphorylation at Serine 194 in animal models. There are several kinases capable of phosphorylating FADD, e.g. CK1α 24, PLK1 25, FIST/HIPK3 26, PKCζ 27, and Aur-A 28, among which CK1α and PLK1 can phosphorylate FADD at Serine 194. As for de-phosphorylation at Serine 194 of FADD, it is mediated by AK2/DUSP26 complex^29^. Therefore, we attempted to search for SNPs associated with both ALL and these kinases. 8, 19, 16 and 10 SNPs were found to be related to the upstream kinases, i.e. CK1α, PLK1, AK2, DUSP26, respectively. Detailed information about these SNPs is provided in Table 2-5. As a result, SNP analysis provided evidence that FADD is linked to ALL susceptibility and is worth investigating.

### 3.2 FADD knockout sensitized Jurkat cells to Etoposide

**Figure 2:**
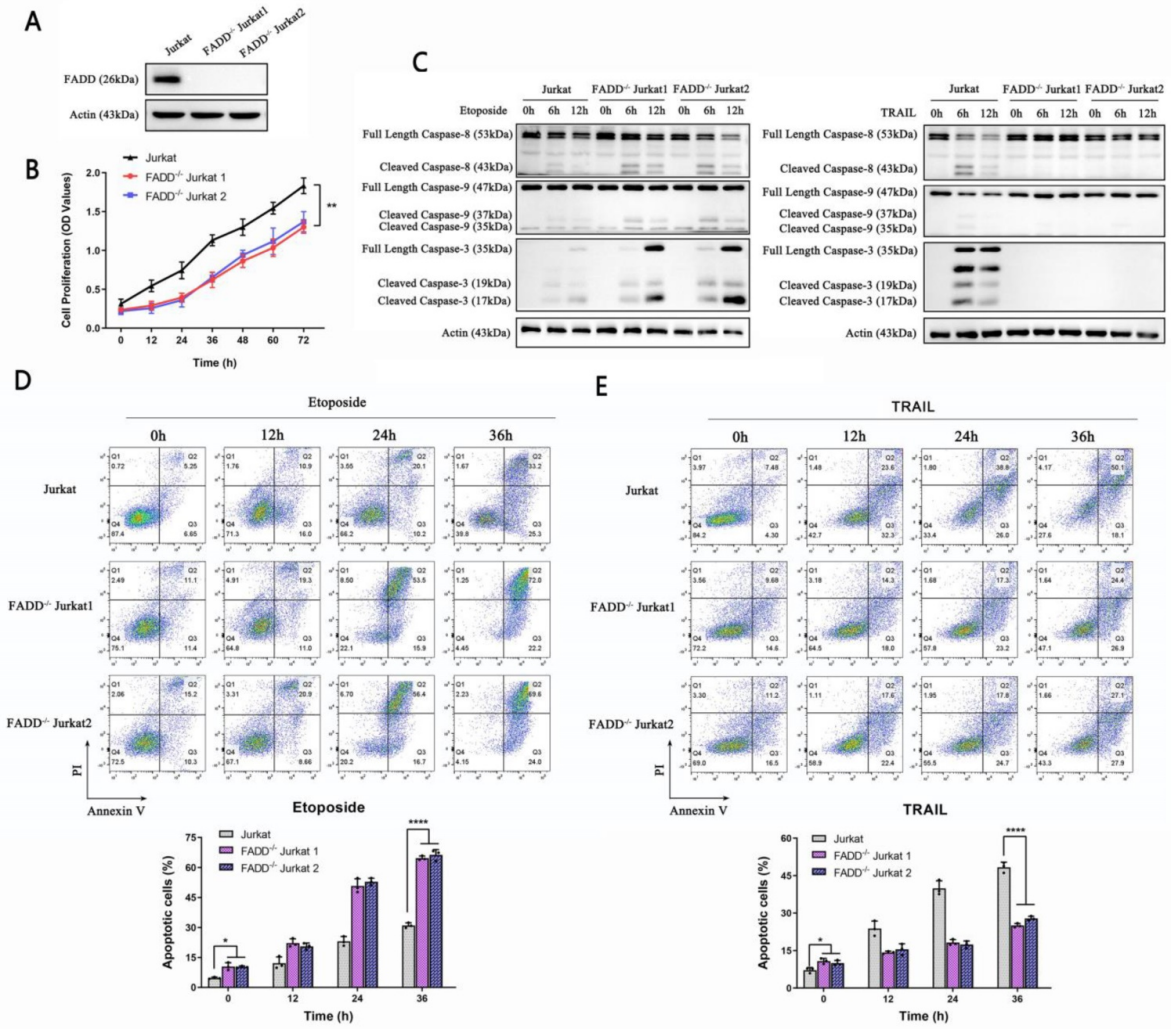
Stable depletion of FADD sensitized Jurkat cells to Etoposide. A. Confirmation of FADD knockout by immunoblot. As mentioned in the Methods and Materials section, FADD^-/-^ Jurkat cells were constructed using two sgRNA sequences to avoid off-target effect. B. CCK-8 proliferation assay of wild type or FADD^-/-^ Jurkat cells. C. Immunoblot of Caspase 3, 8, and 9, following Etoposide or TRAIL treatment to either wild type or FADD^-/-^ Jurkat cells. D. Annexin V/PI double staining monitored by flow cytometry of cells treated by Etoposide. A quantitative analysis is in the lower half of this graph. E. Annexin V/PI double staining monitored by flow cytometry of cells treated by TRAIL. A quantitative analysis is in the lower half of this graph.

FADD is an adaptor protein critical for commencing apoptosis; therefore one may expect certain degree of resistance to apoptosis upon FADD's loss. We constructed Jurkat cells stably depleted of FADD expression using lentivirus. To avoid off-target effects, we constructed two FADD^-/-^ Jurkat cell lines, each with a different sgRNA sequence. FADD knockout was confirmed by western blot (Figure 2A). CCK-8 proliferation assay revealed inhibited proliferative potential of FADD^-/-^ cells (Figure 2B). Apoptosis-related experiments unraveled interesting features. FADD loss endowed Jurkat cells with resistance to TRAIL-induced extrinsic apoptosis as weak cleaved caspase-8 expression along with undetected caspase 3 expression (Figure 2C right panel). In contrast, FADD loss sensitized Jurkat cells to Etoposide-induced intrinsic apoptosis as both cleaved-caspase 9 and caspase 3 expression elevated (Figure 2C left panel). Further confirmation by flow cytometry showed similar results in Figure 2D and E. FADD loss increased the degree of apoptosis induced by Etoposide, whereas substantially inhibited apoptosis induced by TRAIL.

### 3.3 Proteomic analysis

**Figure 3:**
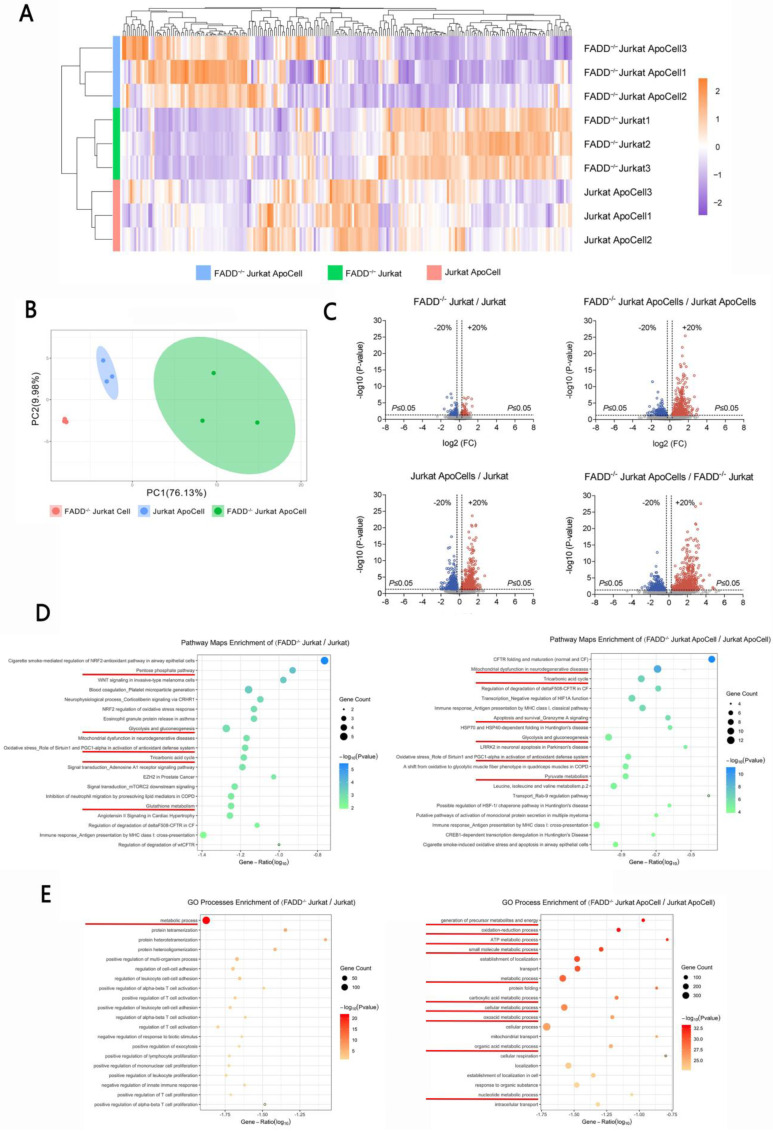
iTRAQ-based proteomics revealed vastly altered spectrum of gene expression upon FADD loss in Jurkat cells. A. Heat maps of differentially expressed genes. ApoCell stands for apoptotic cells, i.e. cells subjected to apoptotic treatment. B. Principal component analysis of the proteome data from different samples. C. Volcano plots using a threshold of <0.8 or >1.2, p-value cutoff is 0.05. Comparison is indicated on the top of each plot D. Pathway maps enrichment generated by MetaCore. Comparison is indicated on the top of each graph. Pathways related to glycolysis or mitochondrial respiration are underlined. E. Gene Ontology enrichment generated by MetaCore. Comparison is indicated on the top of each graph. Processes related to glycolysis or mitochondrial respiration are underlined.

Because FADD loss sensitized FADD^-/-^ cells to Etoposide, we intended to unveil the underlying causes using approaches of proteomics. Four samples were prepared, each in triplicates, for iTRAQ-based comparative proteomics. These samples are wild type Jurkat, apoptotic wild type Jurkat, FADD^-/-^ Jurkat, and apoptotic FADD^-/-^ Jurkat (apoptosis induced by Etoposide). Figure 3A shows a heatmap of different samples. The default, which was wild type Jurkat to which other samples were normalized, is not shown in Figure 3A. It is evident that FADD loss induced a broad change of gene expressions, both in normal and apoptotic samples. Figure 3B shows the Principal Component Analysis (PCA) of our samples. Well separated clusters, with each cluster corresponding to a different treatment group (sample), indicate each sample had a unique pattern of gene expression. Besides, the closer dots of the same color cluster, the better the reproducibility of each sample. Figure 3C shows the volcano plots from pair-wise comparisons. Comparisons were made between untreated cells (upper left), apoptotic cells (upper right), wild type apoptotic vs. untreated cells, and FADD^-/-^ apoptotic and untreated cells. All four comparisons showed vast difference in gene expression pattern. Figure 3D is MetaCore-generated pathway maps enrichment for two comparisons, FADD^-/-^ vs. wild type Jurkat cells and apoptotic FADD^-/-^ vs. wild type Jurkat cells. Figure 3E is MetaCore-generated Gene Ontology (GO) enrichment of the same two comparisons. In pathway maps, we discovered multiple pathways related to metabolism, e.g. glycolysis and gluconeogenesis, tricarboxylic acid cycle and pyruvate metabolism. Metabolism-related pathways are underlined in Figure 3D. Similarly, GO processes enrichment analysis include multiple metabolic processes in Figure 3E. Therefore, based on proteomic analysis, we believe FADD has a role in metabolism in Jurkat cells. To elaborate, FADD loss may effect a shift from glycolysis to aerobic respiration in Jurkat cells.

### 3.4 FADD loss decreased glycolytic capacity in Jurkat cells

**Figure 4:**
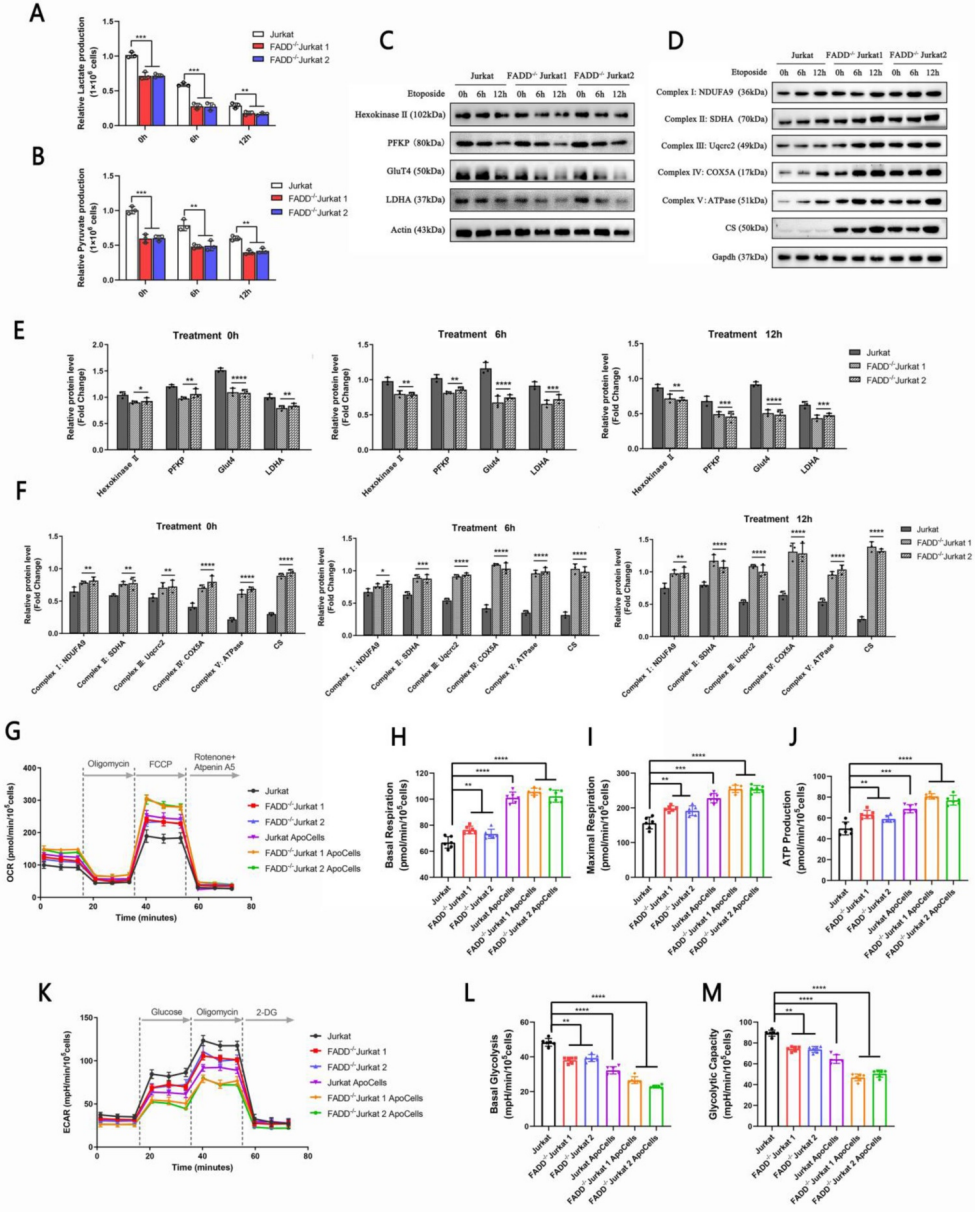
FADD initiated a metabolic shift from glycolysis to mitochondrial respiration. A. Lactate production assay. B. Pyruvate production assay. C. Immunoblots of glycolytic enzymes. D. Immunoblots of enzymes involved in mitochondrial respiration. E. Quantitative analysis of Figure 4C. F. Quantitative analysis of Figure 4D. G. Oxidative phosphorylation profile of samples by measuring OCR before and following injections of oligomycin, FCCP, antimycin A and rotenone. Basal respiration (H), maximal respiration(I) and ATP production (J) in corresponding samples. K. Glycolytic stress profile of samples by measuring ECAR before and following injections of glucose, oligomycin and 2-DG at the time points indicated. Basal (L) and maximal(M) glycolytic capacity in corresponding samples.

Because proteomic analysis revealed changes in both mitochondrion-related and glycolysis-related pathways upon FADD loss, we attempted to probe glycolysis and mitochondrial respiration-related parameters. Figure 4A and B show lactate and pyruvate production in wild type and FADD^-/-^ Jurkat cells subjected to Etoposide treatment. For untreated cells, FADD loss decreased both lactate and pyruvate production levels. The same is true for apoptotic cells. We further detected protein levels of key enzymes in glycolysis and mitochondrial respiration. Immunoblots of certain enzymes in glycolysis, i.e. Hexokinase II (HK2), Phosphofructokinase (Platelet type) (PFKP), Glucose Transporter 4 (GluT4), and Lactate dehydrogenase A (LDHA), were performed (Figure 4C). For untreated cells, protein levels of all glycolytic enzymes were higher in wild type Jurkat than its FADD^-/-^ counterparts. Again, protein levels of these enzymes were still lower in FADD^-/-^ cells at 6 h and 12 h after Etoposide treatment compared to wild type Jurkat cells. Quantitative analysis of immunoblots are shown in Figure 4F. Immunoblots of essential enzymes in the respiratory chain were also performed (Figure 4D). Protein levels of complex I-V, and another key enzyme in the TCA cycle, Citrate Synthase (CS), were indeed higher in FADD^-/-^ cells than wild type cells, and their expressions remained higher at all monitored points of time during Etoposide treatment. Quantitative analysis of Figure 4D is shown in Figure 4F.

We then probed expressions of key enzymes in mitochondrial respiration. In Figure 4D, protein levels of complex I-V and CS were shown for different treatment groups. It is evident that, for untreated cells, expression levels of complex I-V and CS were all higher in FADD^-/-^ cells than wild type cells. Upon apoptotic treatment, expression levels of these enzymes in fact rose higher compared to untreated cells, nevertheless those in FADD^-/-^ cells were still higher than wild type cells. Quantitative analysis of protein levels of these mitochondrial enzymes is shown in Figure 4F.

Another type of assay, using the Agilent Seahorse XF Analyzers, revealed a metabolic shift from aerobic glycolysis to mitochondrial respiration in Jurkat cells upon FADD loss. In this assay, Oxygen consumption rates (OCR) and extracellular acidification rates (ECAR) were measured. OCRs within the time interval between the addition of FCCP and Rotenone + Aptenin A5 indicate maximal respiratory capacity, while ECARs within the time interval between addition of oligomycin and 2-DG indicates maximal glycolytic capacity (OCR in Figure 4G and ECAR in Figure 4K). OCRs within the time interval between the addition of oligomycin and FCCP indicate ATP production and those before any additions indicate basal respiration. ECARs within the time interval between the addition of glucose and Oligomycin indicate basal glycolytic capacity while those within the time interval between the addition of Oligomycin and 2-DG indicate glycolytic capacity (maximal glycolysis). Quantitative analyses of basal respiration, maximal respiration, ATP production, basal glycolytic capacity, maximal glycolytic capacity are shown in Figure 4H-M, respectively. To conclude, FADD^-/-^ cells showed higher maximal respiratory capacity but lower maximal glycolytic capacity than their wild type counterparts. Therefore, according to our results, FADD loss effected an obvious metabolic shift towards mitochondrial respiration.

### 3.5 Mitochondrion is substantially influenced by FADD loss in Jurkat cells

**Figure 5:**
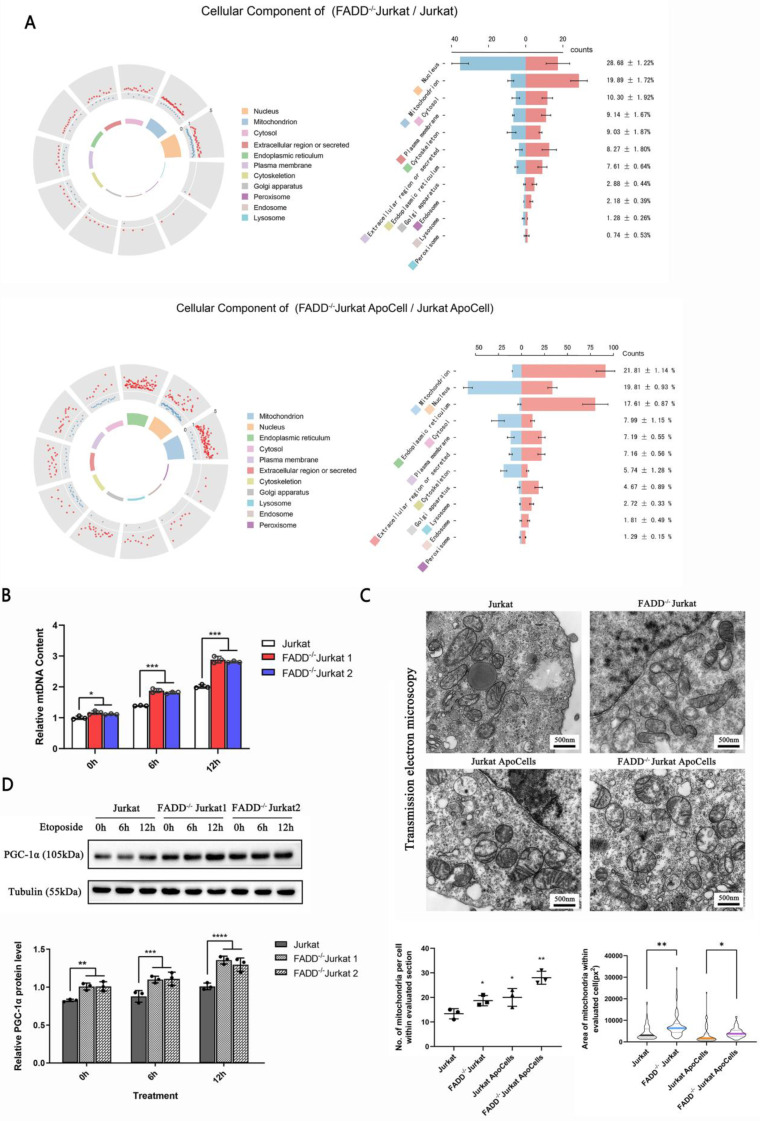
FADD loss altered biology of mitochondria. A. Subcellular localizations of differentially expressed proteins upon FADD loss in Jurkat cells. B. Mitochondrial DNA content detected by qPCR. C. Transmission electron microscopy showing mitochondria in different cells. Quantitative analyses of mitochondria amount and size are in the lower half of this graph. D. Immunoblot of PGC-1α.

We further pursued an analysis of subcellular localizations of all differentially expressed proteins upon FADD loss (fold change cutoff > 20%). Subcellular localizations were obtained and compiled from Uniprot.org using Python. A graph of polar coordinates was produced (Figure 5A). For differentially expressed proteins, two comparisons were made: FADD^-/-^ *vs.* wild type Jurkat cells (Figure 5A upper) and apoptotic FADD^-/-^ *vs.* wild type Jurkat cells (Figure 5A lower). In the group of FADD^-/-^ *vs.* wild type Jurkat cells, mitochondrion appeared to harbor 2^nd^ most differentially expressed proteins upon FADD loss, only fewer than the nucleus. In the group of apoptotic FADD^-/-^ *vs.* wild type Jurkat cells, mitochondrion appeared to harbor most differentially expressed proteins, which explains increased vulnerability to mitochondrion-mediated intrinsic apoptosis upon FADD loss. The right column of Figure 5A shows more detailed statistics of differentially expressed proteins. Blue and red bars in the middle of the two graphs indicate numbers of down and up-regulated proteins in each organelle, respectively. Numbers on the right are proportion of that specific number of differentially expressed proteins in that type of organelle to total number of differentially expressed proteins.

Figure 5B shows relative number of mitochondria based on mitochondrial DNA quantification. Surprisingly, mitochondria in FADD^-/-^ cells outnumbered those in wild type cells, and this might explain why mitochondrial enzymes were up-regulated upon FADD loss. We tried to visualize mitochondria using transmission electron microscopy (Figure 5C). FADD^-/-^ cells appeared to house more and bigger mitochondria than their wild type counterparts, and quantitative analysis of these images is shown in the lower half of Figure 5C. Another protein, PGC-1α, is a positive indicator of mitochondrial functionality. We then probed PGC-1α level in different cell lines (Figure 5D). Untreated FADD^-/-^ cells exhibited higher level of PGC-1α compared to wild type cells, and the same is true for apoptotic FADD^-/-^ cells. A quantitative analysis of PGC-1α is shown in lower half of Figure 5D.

### 3.6 VDAC1 expression was increased upon FADD loss in Jurkat cells

**Figure 6:**
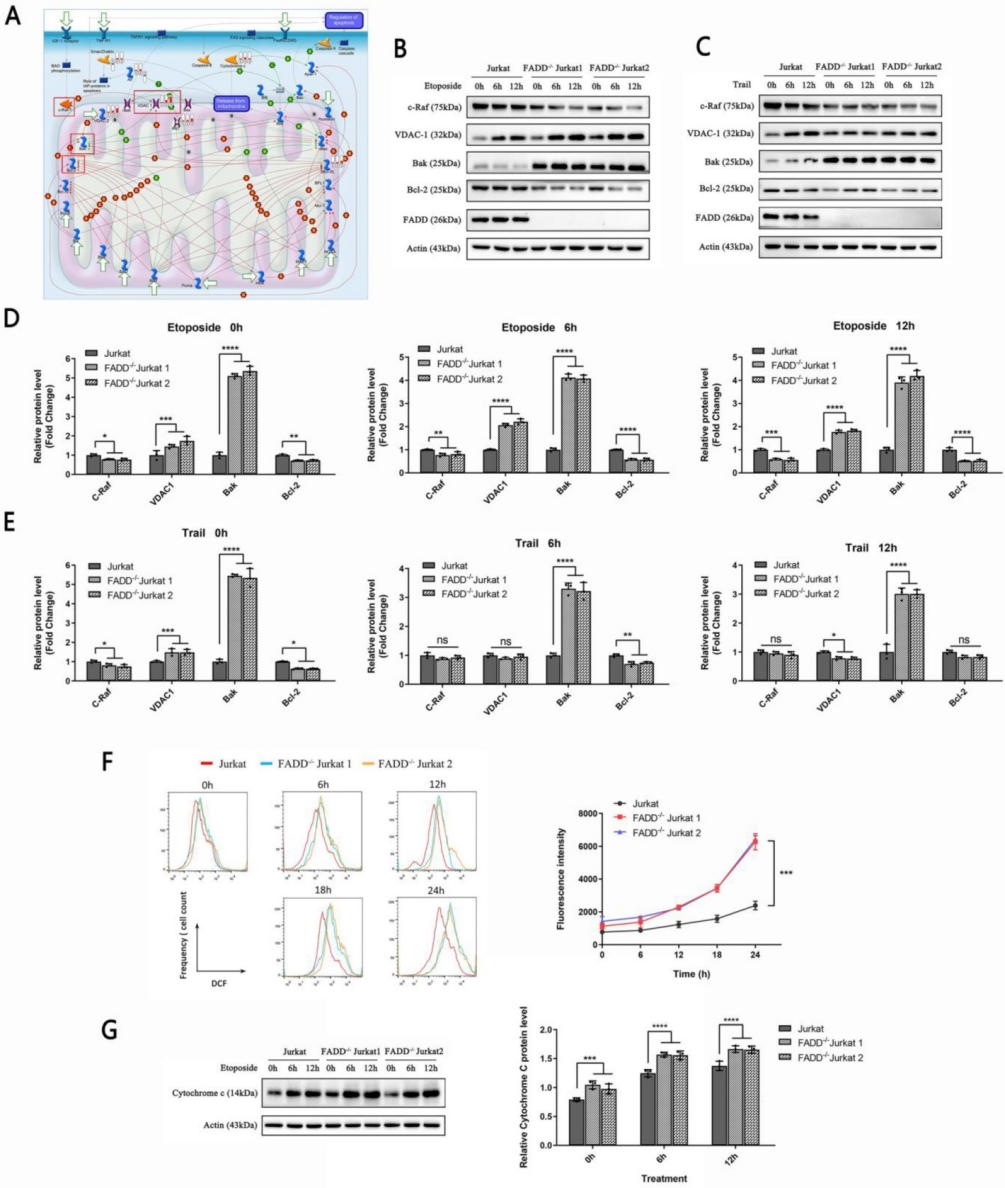
FADD loss increased VDAC1 expression. A. A pathway map entitled Apoptosis and survival regulation of Apoptosis by Mitochondrial Proteins generated by MetaCore. B. Immunoblots of VDAC1, c-Raf, Bak, and Bcl-2 of cells treated by Etoposide. C. Immunoblots of VDAC1, c-Raf, Bak, and Bcl-2 of cells treated by TRAIL. D. Quantitative analysis of Figure 6B. E. Quantitative analysis of Figure 6C. F. ROS production monitored by flow cytometry of cells treated by Etoposide. The right half is a quantitative analysis of the histograms. G. Immunoblots of cytochrome c of cells treated by Etoposide. The bar graphs are quantitative analysis of the immunoblots.

To further investigate mitochondrion-related alterations upon FADD loss, we scrutinized pathway maps generated by MetaCore. We focused on the pathway map entitled Apoptosis and survival regulation of Apoptosis by Mitochondrial Proteins (Figure 6A). Red meters next to certain proteins indicate degree of up-regulation compared to wild type Jurkat cells. Blue meters indicate degree of down-regulation. The meters are shown in the order of FADD^-/-^ Jurkat cells, apoptotic wild type Jurkat cells, and apoptotic FADD^-/-^ Jurkat cells. According to the pathway map, we observed up-regulation of VDAC1 in untreated FADD^-/-^ cell and apoptotic FADD^-/-^ cells. VDAC1 is a pore-forming protein on the mitochondrial membrane. The number of VDAC1 in mitochondrial membrane directly dictates ease of trafficking through the mitochondrial membrane 30. We selected certain proteins shown in the pathway map to validate by immunoblot. These proteins are indicated by red boxes in Figure 6A. c-Raf is a negative regulator of VDAC1, while Bak and Bcl-2 are two members of the Bcl-2 protein family and serve pro- and anti-apoptotic functions, respectively.

We then probed expressions of indicated proteins in Figure 6A by western blot (Figure 6B and C). We observed heightened expressions of VDAC1 and Bak, a pro-apoptotic mitochondrial protein, in untreated FADD^-/-^ cells compared to wild type Jurkat (lane 1, 4, 7 in Figure 6B and C). Upon Etoposide treatment, VDAC1 expression was profoundly increased in both wild type and FADD^-/-^ Jurkat cells (Figure 6B). For Bak expression, increase due to apoptosis was not so obvious in FADD^-/-^ cells, and a slight decrease was observed in wild type cells. The other two proteins, c-Raf and Bcl-2, displayed an opposite trend. c-Raf and Bcl-2 expressions were indeed higher in untreated wild type Jurkat cells than in FADD^-/-^ cells. Etoposide treatment decreased expression of these two proteins in both wild type and FADD^-/-^ cells. For TRAIL treatment, VDAC1 and Bak expressions were increased upon apoptosis while c-Raf and Bcl-2 expressions were decreased in wild type Jurkat cells. Because FADD^-/-^ Jurkat cells are highly resistant to TRAIL-induce apoptosis, no discernible changes were observed for all four proteins upon TRAIL treatment in FADD^-/-^ Jurkat cells. FADD expression was not detected in two FADD^-/-^ cell lines due to FADD knockout. Quantitative analysis of Figure 6B and C are shown in Figure D and E.

Reports have linked increased VDAC1 expression to heightened ROS production 31. We therefore monitored ROS level in FADD^-/-^ cells. At various time points of the apoptotic treatment by Etoposide, ROS level was monitored by flow cytometry in three cell lines (Figure 6H). At all points of time during the apoptotic treatment, ROS level in FADD^-/-^ cells was indeed higher than that in the wild type counterpart. Furthermore, ROS level was positively correlated with length of apoptotic treatment as ROS accumulated as apoptosis proceeded further.

VDAC1 expression is also connected to cytochrome c expression 30. Therefore, we probed cytochrome c expression in three cell lines subjected to Etoposide treatment (Figure 6I). For untreated cells, FADD^-/-^ cells showed higher basal levels of cytochrome c than wild type Jurkat cells. As apoptosis deepened, cytochrome c expression further increased in all three cell lines. At each point of time during the apoptotic treatment, FADD^-/-^ cells showed higher protein level of cytochrome c than wild type Jurkat cells.

### 3.7 FADD knock-in partially rescued oncogenicity in FADD^-/-^ Jurkat

**Figure 7:**
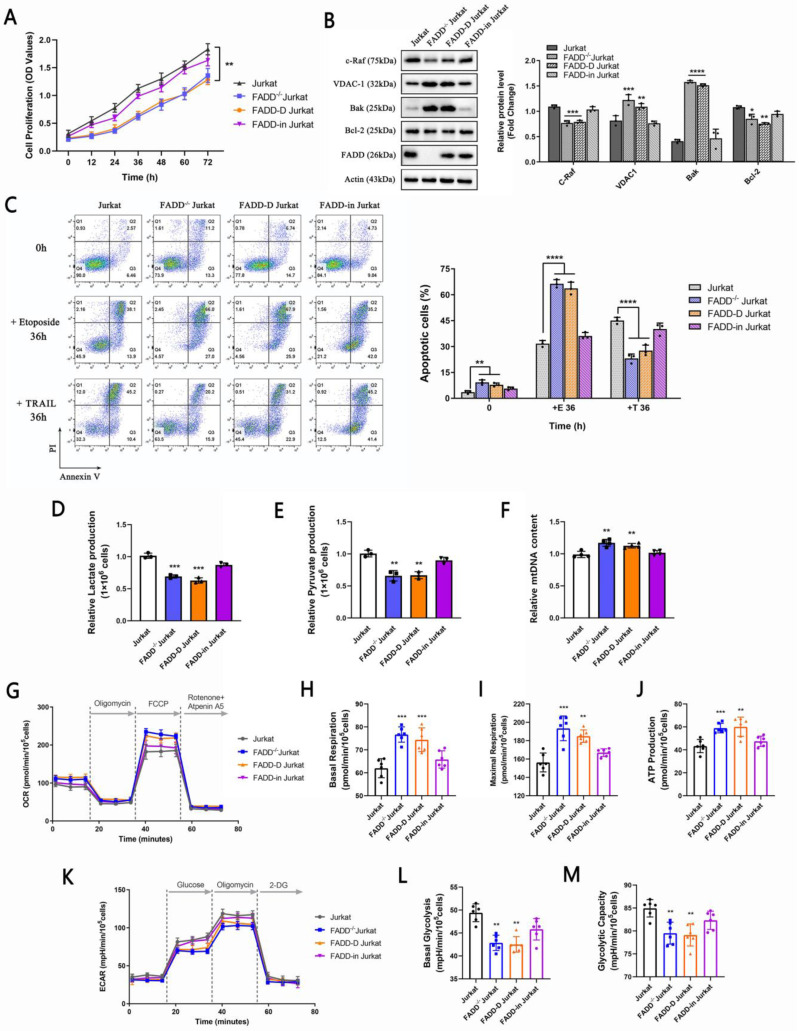
Re-expression of FADD partially restored oncogenicity in Jurkat cells. A.CCK-8 cell proliferation assay. FADD-D indicates FADD^-/-^ cells stably expressing FADD-D. FADD-in indicates FADD^-/-^ cells stably expressing wild type FADD. B. Immunoblots of VDAC1, c-Raf, Bak, and Bcl2. The bar graphs are quantitative analysis of the blots. C. Annexin V/PI double staining of various cell lines subjected to Etoposide treatment. The bar graphs are quantitative analysis. D. Lactate production assay. E. Pyruvate production assay. F. mitochondrial DNA content. G. Oxidative phosphorylation profile of samples by measuring OCR before and following injections of oligomycin, FCCP, antimycin A and rotenone. Basal respiration (H), maximal respiration (I) and ATP production (J) in corresponding samples. K. Glycolytic stress profile of samples by measuring ECAR before and following injections of glucose, oligomycin and 2-DG at the time points indicated. Basal (L) and maximal (M) glycolytic capacity in corresponding samples.

To validate tumor-suppressive effects of FADD loss (or oncogenic potential of FADD), we performed rescue experiments by overexpressing FADD or FADD-D (Serine 194 permanently replaced by Asparagine, mimicking pFADD which is a phosphorylated form of FADD at Serine 194) in FADD^-/-^ Jurkat cells. There are reports which connect the level of pFADD to poor clinical outcomes 29, 32-34. Inclusion of FADD-D in our rescue experiments aims to unravel the role of FADD-D in Jurkat cells. Figure 7A shows proliferation profiles of different cells. Noticeably, wild type and FADD-in Jurkat cells (FADD^-/-^ cells expressing plasmids harboring wild type FADD ORF) displayed similar and relatively high rates of proliferation as determined by CCK-8 assay, indicative of restoration of oncogenicity by FADD re-expression in FADD^-/-^ cells. On the other hand, FADD^-/-^ and FADD-D Jurkat cells (FADD^-/-^ cells stably expressing FADD-D) displayed similar and low rates of proliferation, indicative of inability of FADD-D to restore oncogenicity.

Protein expression levels of certain mitochondrial proteins were also monitored. As expected, expressions of the proto-oncogene c-Raf and anti-apoptotic factor Bcl-2 were higher in wild type and FADD-in Jurkat cells, while expressions of VDAC1 and Bak were higher in FADD^-/-^ and FADD-D Jurkat cells. Immunoblot results were in agreement with proliferation rates monitored by CCK-8 assay. Re-expression of wild type FADD partially restored expressions of c-Raf and Bcl-2 whereas re-expression of FADD-D failed to achieve so.

Wild type and FADD-in Jurkat cells were more resistant to Etoposide-induced apoptosis than FADD^-/-^ and FADD-D Jurkat cells (Figure 7C). On the contrary, FADD^-/-^ and FADD-D Jurkat cells were more resistant to TRAIL-induced apoptosis. Likewise, Results from apoptosis treatments were in accordance with previous results and therefore further consolidate the finding that FADD is likely an oncogenic protein which confers resistance to Etoposide in Jurkat cells.

Glycolytic parameters and mtDNA content were also monitored, i.e. lactate and pyruvate production (Figure 7D-F). Wild type and FADD-in Jurkat cells produced similar amount of lactate and pyruvate, suggesting restored glycolytic capacity by re-expression of wild type FADD. FADD^-/-^ and FADD-D Jurkat cells didn't produce as much lactate and pyruvate, suggesting inability of FADD-D to rescue glycolytic capacity in Jurkat cells. mtDNA content exhibited similar results.

Lastly, we monitored OCRs and ECARs of different cell lines (Figure 7G-M). Evidently, wild type and FADD-in Jurkat cells showed higher values in both basal and maximal respiration as well as ATP production compared to FADD^-/-^ and FADD-D Jurkat cells. In contrast, FADD^-/-^ and FADD-D Jurkat cells showed higher values in both basal and maximal respiration as well as ATP production compared to wild type and FADD-in Jurkat cells. Thus, reconstitution of FADD in FADD^-/-^ cells indeed rescued glycolytic capacity in Jurkat cells while re-expression of FADD-D, a phosphorylated form, did not accomplish such effect.

## Discussion

The most prominent feature of cancer has long been considered to be uncontrolled growth. The traditional six hallmarks of cancer all seemingly to be unrelated to metabolism 13. However, evidence in the last decade has linked the distinctive metabolism of cancer to its invasive features. Reliance on glycolysis appears to be a non-ideal strategy for cancer cells in terms of energetics, yet it confers cancer cells other advantages such as acidic microenvironment 35, 36. Lactate produced by glycolysis in cancer causes acidosis, and acidification of the tumor microenvironment is now considered to be a direct consequence of the Warburg effect. The acidic tumor microenvironment then alters a spectrum of gene expressions favoring cancer progression and malignancy 37.

The FADD protein, which was regarded as a regulator of life and death, has now been considered as a multi-functional protein. FADD's non-apoptotic functions cover a wide range of biological processes. Its involvements in lipid metabolism and glucose uptake have both been reported. Yet its role in cancer has not been systematically studied.

Our aim was to pinpoint FADD's role. We attempted to seek connections between FADD and ALL using bioinformatics. Although SNPs are the most common genetic variations among human beings, they can also lead to diseases. Multiple reports have connected SNPs to ALL 38, 39. We performed a SNP analysis and discovered 17 SNPs which are associated with both FADD expression and ALL susceptibility 40. Resistance to the TRAIL-induced extrinsic apoptosis in FADD^-/-^ Jurkat cells were observed and was indeed expected because the extrinsic apoptotic pathway was severed due to loss of FADD in FADD^-/-^ cells. However, increased vulnerability to Etoposide seemingly contradicts FADD's role being as pivotal factor in apoptosis induction. Analysis of proteomics data by MetaCore revealed altered pathways in mitochondrial respiration and glycolysis. By monitoring relevant parameters and enzyme expressions, it was evident that FADD loss caused a metabolic shift towards mitochondrial respiration, diminishing the strength of glycolysis in Jurkat cells. Therefore, a direct link between FADD and glycolysis in Jurkat cell was discovered.

Other pieces of information obtained from MetaCore include increased expression of the mitochondrial pore-forming protein, VDAC1, in FADD^-/-^ Jurkat cells. Increased VDAC1 expression leads to increased number of channels/pores on the mitochondrial membrane and therefore elevated trafficking of materials through the mitochondrial membrane. Upon intrinsic apoptosis, the enhanced propensity of cytochrome c release due to increased number of pores is the direct reason for increased sensitivity of FADD^-/-^ cells to Etoposide. Furthermore, elevation in ROS was also observed in FADD^-/-^ Jurkat cells. ROS is a subtle companion of cancer as too much or too little ROS both hinder cancer progression. As a byproduct of ATP synthesis in the process of oxidative phosphorylation, ROS generation is necessary for energy production. However, cancer cells devise a delicate balance between aerobic respiration and glycolysis to circumvent negative effects exerted by excessive ROS produced by oxidative phosphorylation. FADD knockout increased ROS generation by increasing VDAC1 expression, and maybe it alters the balance between aerobic respiration and glycolysis. This alteration of metabolism balance eventually caused a shift towards aerobic respiration, and therefore deprived the advantages conferred by reliance on glycolysis for tumor cells.

Reconstitution experiments by re-expressing FADD or FADD-D in FADD^-/-^ Jurkat cells showed that, FADD but not FADD-D, partially rescued glycolytic capacity in Jurkat cells and therefore restored certain degree of oncogenicity. FADD-D failed to restore glycolytic capacity. As mentioned before, increases and decreases in pFADD have both been observed in cancer. As our results dictate, pFADD does not appear to function as an oncogenic protein in Jurkat cells.

**Figure 8:**
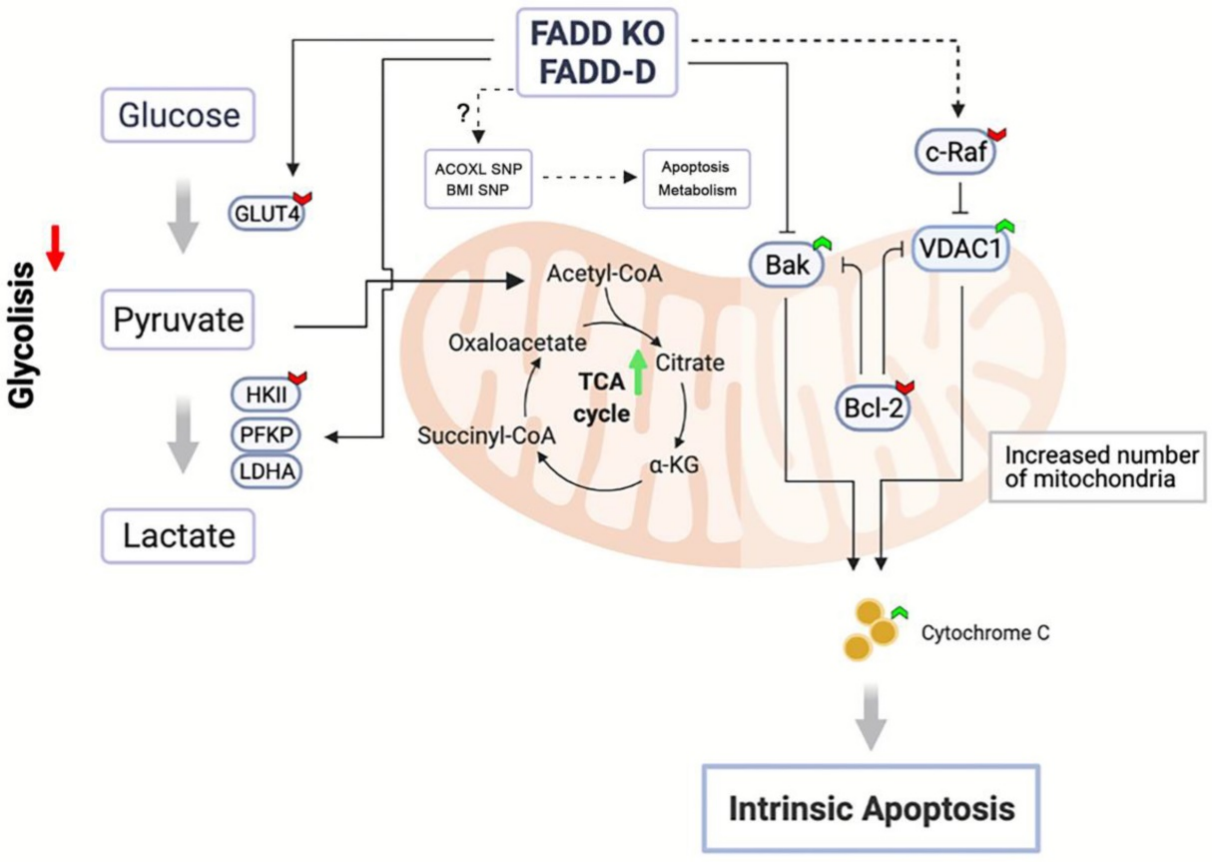
Schematic representation of FADD-induced metabolic shift towards mitochondrial respiration.

Figure 8 illustrates our conclusions. Upon FADD loss or increase in FADD phosphorylation, SNP status of multiple genes may be altered in certain ways to effect changes in metabolism according to bioinformatics, e.g. SNP of ACOXL is related to lipid metabolism. Furthermore, our results showed that glycolysis in Jurkat cells is dampened and enzyme expressions of the glycolytic pathway (HK2, PFKP, and LDHA) are reduced upon FADD loss or phosphorylation. On the other hand, enzymes associated with mitochondrial respiration are enhanced. VDAC1 and Bak expressions are elevated while c-Raf and Bcl-2 expressions are lessened. Increased VDAC1 expressions lead to easier release of cytochrome c and thus increased susceptibility to Etoposide. Our study revealed, for the first time, a regulatory and non-apoptotic role of FADD and its phosphorylated form in metabolic reprogramming ALL using Jurkat cells as a model. FADD expression level is potentially an influencing factor when administrating chemotherapy and therefore could be interfered to enhance chemotherapeutic efficacy.

## Supplementary Material

Supplementary tables.

## Figures and Tables

**Table 1:** Detailed information of ALL-associated SNPs (whole blood samples) which are also related to FADD expression

No	rsID	P_leu_	P_FADD_	Mapped gene	Mapped Gene-GO Biological process terms/keywords
1	rs72926690	3 x 10^-6^	0.000079	AL359987.1, LINC02531	uncharacterized protein
2	rs17483466	2 x 10^-10^	0.0054	ACOXL, MIR4435-2HG	fatty acid beta-oxidation using acyl-CoA oxidase, lipid homeostasis, fatty acid beta-oxidation, oxidation-reduction process
		5 x 10^-9^			
		4 x 10^-17^			
3	rs7578199	8 x 10^-8^	0.012	HDLBP	cholesterol metabolic process, lipid metabolic process, lipid transport, high-density lipoprotein particle clearance
4	rs806321	2 x 10^-8^	0.019	DLEU1	no Annotation
5	rs73005220	1 x 10^-6^	0.02	CIB3	no Annotation
6	rs2466024	3 x 10^-6^	0.025	PCAT1, CASC19	no Annotation
7	rs35603048	4 x 10^-7^	0.025	BMF	apoptosis
8	rs58055674	2 x 10^-27^	0.027	MIR4435-2HG, ACOXL	fatty acid beta-oxidation using acyl-CoA oxidase, lipid homeostasis, fatty acid beta-oxidation, oxidation-reduction process
		5 x 10^-20^			
9	rs12711846	3 x 10^-14^	0.029	MIR4435-2HG, ACOXL, ACOXL-AS1	
10	rs138922423	3 x 10^-6^	0.032	LRRIQ3, AL591463.1	no Annotation
11	rs142811167	5 x 10^-6^	0.037	OVCH2	proteolysis
12	rs1439112	7 x 10^-9^	0.04	MGAT5	positive regulation of receptor signaling pathway via STAT, positive regulation of cell migration, negative regulation of protein tyrosine phosphatase activity, protein N-linked glycosylation via asparagine, protein phosphopantetheinylation
13	rs898518	4 x 10^-10^	0.04	LEF1	Transcription regulation, Wnt signaling pathway
14	rs7677291	4 x 10^-6^	0.043	AC082650.1, AC105417.1	no Annotation
15	rs17134658	5 x 10^-6^	0.048	CNTN5	cell adhesion, presynapse assembly, sensory perception of sound
16	rs12530134	4 x 10^-6^	0.049	AL031259.1, PDCD2	Apoptosis
17	rs1805465	6 x 10^-6^	0.049	MIS12	Cell cycle, Cell division, Chromosome partition, Mitosis

Note: P_leu_: P value for association with locus and leukemia susceptibility. P_FADD_: P value for association with locus and FADD gene expression.

**Table 2:** Detailed information of ALL-associated SNPs (whole blood samples) which are also related to CK1α expression

No	rsID	P_leu_	P_CK1α_	Mapped gene	Mapped Gene-GO Biological process terms/keywords
1	rs73195662	1 x 10^-6^	0.0092	CDHR3	Cell adhesion, Host-virus interaction
2	rs67134687	4 x 10^-6^	0.028	MTAP, AL359922.1	response to testosterone, L-methionine salvage from methylthioadenosine, nicotinamide riboside catabolic process, nucleoside metabolic process, interleukin-12-mediated signaling pathway
3	rs2267708	9 x 10^-9^	0.038	GPR37	G protein-coupled receptor signaling pathway, locomotion involved in locomotory behavior, negative regulation of hydrogen peroxide-induced cell death, positive regulation of dopamine metabolic process, positive regulation of MAPK cascade
4	rs17007695	9 x 10^-7^	0.039	IL15, INPP4B	IL15: macrophage differentiation, positive regulation of immune response, neutrophil activation, positive regulation of cell population proliferation, aging, skeletal muscle atrophy, hyaluronan metabolic process, cellular response to vitamin D, regulation of T cell differentiation, positive regulation of protein O-linked glycosylation, response to nutrient levels, positive regulation of natural killer cell differentiation, lymph node development, negative regulation of cold-induced thermogenesis, positive regulation of tissue remodeling, positive regulation of peptidyl-tyrosine phosphorylation, positive regulation of phagocytosis INPP4B: signal transduction, phosphatidylinositol biosynthetic process, inositol phosphate metabolic process, phosphatidylinositol-3-phosphate biosynthetic process
5	rs3130284	7 x 10^-6^	0.042	AGPAT1	Lipid biosynthesis, Lipid metabolism, Phospholipid biosynthesis, Phospholipid metabolism
6	rs3096696	4 x 10^-6^	0.042	PPT2, PPT2-EGFL8	fatty-acyl-CoA biosynthetic process, macromolecule depalmitoylation, protein phosphopantetheinylation
7	rs117483095	4 x 10^-6^	0.044	FNBP1	signal transduction, endocytosis, membrane organization

Note: P_leu_: P value for association with locus and leukemia susceptibility. P_CK1α_: P value for association with locus and CK1α gene expression.

**Table 3:** Detailed information of ALL-associated SNPs (whole blood samples) which are also related to PLK1 expression

No	rsID	P_leu_	P_PLK1_	Mapped gene	Mapped Gene-GO Biological process terms/keywords
1	rs2239630	2 x 10^-21^	0.0025	SLC7A8, CEBPE	SLC7A8: Amino-acid transport, Transport CEBPE: Transcription, Transcription regulation
2	rs75777619	2 x 10^-9^	0.003	CCDC26	no Annotation
3	rs28665337	4 x 10^-9^	0.0036		
4	rs6893857	2 x 10^-6^	0.011	ARSB	colon epithelial cell migration, lysosome organization, neutrophil degranulation, chondroitin sulfate catabolic process, response to estrogen, response to pH, response to nutrient, response to methylmercury, positive regulation of neuron projection development, autophagy
5	rs7973974	3 x 10^-6^	0.016	BCAT1	Leucine and valine biosynthetic process, branched-chain amino acid metabolic process, cellular amino acid biosynthetic process, G1/S transition of mitotic cell cycle
6	rs10949482	1 x 10^-20^	0.018	NHLRC1	Autophagy, Ubl conjugation pathway
		1 x 10^-33^			
7	rs12711846	3 x 10^-14^	0.018	MIR4435-2HG, ACOXL, ACOXL-AS1	fatty acid beta-oxidation using acyl-CoA oxidase, lipid homeostasis, fatty acid beta-oxidation, oxidation-reduction process
8	rs17483466	2 x 10^-10^	0.019	ACOXL, MIR4435-2HG	
		5 x 10^-9^			
		4 x 10^-17^			
9	rs58055674	2 x 10^-27^	0.019		
		5 x 10^-20^			
10	rs61610071	2 x 10^-6^	0.02	AL355870.2, SEPHS1	phosphorylation, cellular protein modification process, selenocysteine biosynthetic process
11	rs2239633	1 x 10^-16^	0.021	CEBPE, SLC7A8	CEBPE: phagocytosis, positive regulation of gene expression, macrophage differentiation, defense response to bacterium, positive regulation of transcription by RNA polymerase II, myeloid cell differentiation SLC7A8: leukocyte migration, proline transmembrane transport, valine transport, tryptophan transport, leucine transport, glycine transport, toxin transport, metal ion homeostasis, thyroid hormone transport, transport across blood-brain barrier
		5 x 10^-14^			
		3 x 10^-7^			
		2 x 10^-8^			
		4 x 10^-10^			
		7 x 10^-13^			
12	rs10849033	9 x 10^-6^	0.026	TIGAR, AC008012.1	fructose 2,6-bisphosphate metabolic process, positive regulation of DNA repair, negative regulation of glycolytic process, regulation of pentose-phosphate shunt, cellular response to cobalt ion, response to ischemia, autophagy, negative regulation of reactive oxygen species metabolic process
13	rs4617118	2 x 10^-12^	0.028	CCDC26	no Annotation
14	rs6489882	5 x 10^-8^	0.03	AC004551.1, OAS3	Antiviral defense, Immunity, Innate immunity
15	rs1805465	6 x 10^-6^	0.036	MIS12	Cell cycle, Cell division, Chromosome partition, Mitosis
16	rs1986582	7 x 10^-6^	0.047	GAPDHP68, AC004852.2	no Annotation
17	rs57214277	4 x 10^-8^	0.049	MYL12BP2, LINC02363	no Annotation

Note: P_leu_: P value for association with locus and leukemia susceptibility. P_PLK1_: P value for association with locus and PLK1 gene expression.

**Table 4:** Detailed information of ALL-associated SNPs (whole blood samples) which are also related to AK2 expression

No	rsID	P_leu_	P_AK2_	Mapped gene	Mapped Gene-GO Biological process terms/keywords
1	rs6858698	3 x 10^-9^	0.0036	CAMK2D, AC111193.1	cardiac muscle cell contraction, cellular response to calcium ion, negative regulation of sodium ion transmembrane transport, positive regulation of cardiac muscle cell apoptotic process, positive regulation of cardiac muscle hypertrophy, protein phosphorylation, regulation of histone deacetylase activity, regulation of transcription by RNA polymerase II
2	rs1476569	6 x 10^-10^	0.0062		
3	rs6445754	5 x 10^-6^	0.0051	ERC2	maintenance of presynaptic active zone structure, regulation of synaptic plasticity, synaptic vesicle priming
4	rs735665	4 x 10-^12^	0.01	GRAMD1B	cellular response to cholesterol, cholesterol homeostasis
		3 x 10^-12^			
		4 x 10^-24^			
		4 x 10^-39^			
5	rs35923643	4 x 10^-58^	0.012		
		2 x 10^-40^			
6	rs10849033	9 x 10^-6^	0.013	TIGAR, AC008012.1	fructose 2,6-bisphosphate metabolic process, positive regulation of DNA repair, negative regulation of glycolytic process, regulation of pentose-phosphate shunt, cellular response to cobalt ion, response to ischemia, autophagy, negative regulation of reactive oxygen species metabolic process
7	rs2267708	9 x 10^-9^	0.015	GPR37	G protein-coupled receptor signaling pathway, locomotion involved in locomotory behavior, negative regulation of hydrogen peroxide-induced cell death, positive regulation of dopamine metabolic process, positive regulation of MAPK cascade
8	rs7578199	8 x 10^-8^	0.019	HDLBP	cholesterol metabolic process, lipid transport
9	rs114961115	7 x 10^-7^	0.021	ENPP5, ACTG1P9	cell communication
10	rs4266947	7 x 10^-6^	0.021	LGR6	axon guidance, bone regeneration, negative chemotaxis, positive regulation of cell migration, positive regulation of Wnt signaling pathway
11	rs267759	7 x 10^-6^	0.032	LMBRD2	adrenergic receptor signaling pathway
12	rs630662	2 x 10^-6^	0.034	AC025508.1, RSPO2	Sensory transduction, Wnt signaling pathway
13	rs139996880	6 x 10^-8^	0.04	TERT	cellular response to hypoxia, DNA and double-stranded RNA biosynthetic process, mitochondrion organization, negative regulation of cellular senescence, negative regulation of endothelial cell apoptotic process, positive regulation of angiogenesis, positive regulation of G1/S transition of mitotic cell cycle, positive regulation of glucose import, positive regulation of nitric-oxide synthase activity, positive regulation of protein localization to nucleolus, positive regulation of stem cell proliferation, positive regulation of transdifferentiation, positive regulation of Wnt signaling pathway, response to cadmium ion, telomere maintenance, transcription
14	rs1359742	7 x 10^-9^	0.041	AL157937.1, DMRTA1	Transcription regulation
15	rs7090445	5 x 10^-54^	0.048	ARID5B	tissue development, cellular response to leukemia inhibitory factor, fibroblast migration, nitrogen compound metabolic process, platelet-derived growth factor receptor signaling pathway, positive regulation of DNA-binding transcription factor activity, post-embryonic development, skeletal system morphogenesis
16	rs7818688	4 x 10^-8^	0.048	NDUFAF6	biosynthetic process, mitochondrial respiratory chain complex I assembly

Note: P_leu_: P value for association with locus and leukemia susceptibility. P_AK2_: P value for association with locus and AK2 gene expression.

**Table 5:** Detailed information of ALL-associated SNPs (whole blood samples) which are also related to DUSP26 expression

No	rsID	P_leu_	P_DUSP26_	Mapped gene	Mapped Gene-GO Biological process terms/keywords
1	rs73195662	1 x 10^-6^	0.0033	CDHR3	Cell adhesion, Host-virus interaction
2	rs73718779	2 x 10^-8^	0.0086	SERPINB6	cellular response to osmotic stress, negative regulation of endopeptidase activity, sensory perception of sound
3	rs4459895	2 x 10^-7^	0.011	LPP	cell-cell adhesion
4	rs10949482	1 x 10^-20^	0.02	NHLRC1	Autophagy, Ubl conjugation pathway
		1 x 10^-33^			
5	rs187021028	9 x 10^-9^	0.022	LINC01785	uncharacterized protein
6	rs4525246	3 x 10^-14^	0.025	GRAMD1B	Lipid transport, Transport, cholesterol homeostasis
7	rs79050301	8 x 10^-10^	0.03	TPMT	drug metabolic process, methylation, nucleobase-containing compound metabolic process
8	rs1142345	9 x 10^-61^	0.033		
9	rs1036935	3 x 10^-8^	0.038	AC105227.1, AC105227.2	uncharacterized protein
10	rs7578199	8 x 10^-8^	0.038	HDLBP	cholesterol metabolic process, lipid transport

Note: P_leu_: P value for association with locus and leukemia susceptibility. P_DUSP26_: P value for association with locus and DUSP26 gene expression.

## Abbreviations

SNP: Single Nucleotide Polymorphism
TRAIL: TNF-related apoptosis-inducing ligand
sgRNA: single-guide RNA
GO: gene ontology
ATP: Adenosine triphosphate
VEGF: vascular endothelial-derived growth factor
EGFR: Epidermal Growth Factor Receptor
ROS: reactive oxygen species
HK2: hexokinase 2
PKM2: pyruvate kinase M2 subtype
LDHA: lactate dehydrogenase A
ORF: open reading frame
CRISPR: clustered regularly interspaced short palindromic repeats
