# Supplementary material for: The Classical Apoptotic Adaptor FADD Regulates Glycolytic Capacity in Acute Lymphoblastic Leukemia
Source: Int J Biol Sci. 2022 May 1;18(8):3137–55. doi: 10.7150/ijbs.68016 (PMC9134909; doi:10.7150/ijbs.68016)
Supplement: Supplementary file 1 — Supplementary tables. [file ijbsv18p3137s1.pdf]

Supplementary Table 1. Supplementary information of Table 1

No	rsID	Study accession	Chr	Pos	RA	OA	RAF	OR	CI
1	rs72926690	GCST010463	6	92640153	T	C	-	4.46	[2.38-8.38]
2	rs17483466	GCST000224	2	111039881	G	A	0.2	1.39	[1.25-1.53]
		GCST001570			G	A	0.8	1.43	[1.27-1.61]
		GCST002073			G	A	0.2	1.37	[NR]
3	rs7578199	GCST003468	2	241253433	C	T	0.745	1.1764705	[1.11-1.25]
4	rs806321	GCST008721	13	50267187	T	C	NR	1.0863662	-
5	rs73005220	GCST004099	19	16161878	G	A	NR	-	-
6	rs2466024	GCST002299	8	127175774	A	G	0.41	1.21	[NR]
7	rs35603048	GCST004099	15	40099764	T	C	NR	-	-
8	rs58055674	GCST004146	2	111074216	C	T	NR	1.41	[1.32-1.5]
		GCST003468			C	T	0.173	1.44	[1.33-1.56]
9	rs12711846	GCST004099	2	111098716	G	A	NR	-	-
10	rs138922423	GCST010463	1	73916689	T	TA	-	16.02	[4.99-51.46]
11	rs142811167	GCST010463	11	7698038	T	C	-	87.06	[12.84-590.36]
12	rs1439112	GCST008727	2	134305027	A	G	NR	1.1409012	-
13	rs898518	GCST002073	4	108095668	A	C	0.59	1.2	[1.14-1.27]
14	rs7677291	GCST010463	4	129240194	T	C	-	2.11	[1.53-2.9]
15	rs17134658	GCST010463	11	99911908	G	T	-	5.5555553	[2.63-11.11]
16	rs12530134	GCST004250	6	170610382	A	G	-	1.36	[1.03-1.80]
17	rs1805465	GCST002915	17	5489769	T	C	-	2.339	-

Note: RA: risk allele. OA: other allele. RAF: risk allele frequency in controls. OR: odds ratio. CI: 95% confidence interval of OR.

Supplementary Table 2. Supplementary information of Table 2

No	rsID	Study accession	Chr	Pos	RA	OA	RAF	OR	CI
1	rs73195662	GCST007540	7	106018014	G	C	NR	3.52	[2.12-5.85]
2	rs67134687	GCST010463	9	21845309	G	A	-	7.142857	[3.12-16.67]
3	rs2267708	GCST004146	7	124752458	T	C	NR	1.16	[1.1-1.22]
4	rs17007695	GCST000323	4	141788570	C	T	NR	2.67	[1.53-4.68]
5	rs3130284	GCST002915	6	32172710	C	T	NR	1.464	-
6	rs3096696	GCST002915	6	32154695	A	C	-	1.478	-
7	rs117483095	GCST004250	9	130022496	T	C	-	1.62	[1.05-2.50]

Note: RA: risk allele. OA: other allele. RAF: risk allele frequency in controls. OR: odds ratio. CI: 95% confidence interval of OR.

Supplementary Table 3. Supplementary information of Table 3

No	rsID	Study accession	Chr	Pos	RA	OA	RAF	OR	CI
1	rs2239630	GCST009638	14	23120140	G	A	0.45	1.28	[1.22-1.35]
2	rs75777619	GCST009638	8	129172930	G	A	0.12	1.26	[1.17-1.36]
3	rs28665337	GCST005832	8	129181858	A	C	0.12	1.34	[1.21-1.47]
4	rs6893857	GCST003468	5	78888580	C	T	0.183	1.17	[1.10-1.25]
5	rs7973974	GCST007540	12	24847276	C	T	NR	2.71	[1.78-4.11]
6	rs10949482	GCST007149	6	18121083	T	C	NR	-	-
		GCST007148					NR	-	-
7	rs12711846	GCST004099	2	111098716	G	A	NR	-	-
8	rs17483466	GCST000224	2	111039881	G	A	0.2	1.39	[1.25-1.53]
		GCST001570					0.8	1.43	[1.27-1.61]
		GCST002073					0.2	1.37	[NR]
9	rs58055674	GCST004146	2	111074216	C	T	NR	1.41	[1.32-1.5]
		GCST003468					0.173	1.44	[1.33-1.56]
10	rs61610071	GCST004250	10	13373961	G	A	'-	9.02	[1.31-62.00]
11	rs2239633	GCST002158	14	23119848	A	G	NR	-	-
		GCST005832					NR	1.369863	[1.28-1.45]
		GCST000463					0.52	1.34	[1.22-1.45]
		GCST001320					0.5	1.333333	[1.2-1.49]
		GCST001320					0.5	1.351351	[1.22-1.47]
		GCST005315					NR	1.265823	[NR]
12	rs10849033	GCST000464	12	4315956	A	G	0.02	2.55	[1.60-3.80]
13	rs4617118	GCST005315	8	129143897	G	A	NR	1.28	[1.19-1.37]
14	rs6489882	GCST004146	12	112943571	A	G	NR	1.16	[1.1-1.22]
15	rs1805465	GCST002915	17	5489769	T	C	-	2.339	-
16	rs1986582	GCST005214	7	9391401	C	G	0.12	-	-
17	rs57214277	GCST004146	4	184333619	T	C	0.41	1.13	[1.08-1.18]

Note: RA: risk allele. OA: other allele. RAF: risk allele frequency in controls. OR: odds ratio. CI: 95% confidence interval of OR.

Supplementary Table 4. Supplementary information of Table 4

No	rsID	Study accession	Chr	Pos	RA	OA	RAF	OR	CI
1	rs6858698	GCST002299	4	113762688	C	G	-	1.31	[1.20-1.44]
2	rs1476569	GCST004146	4	113777540	G	A	NR	1.18	[1.12-1.25]
3	rs6445754	GCST001320	3	55773227	T	C	0.22	-	-
4	rs735665	GCST000224	11	123490689	A	G	0.21	1.45	[1.31-1.61]
		GCST001570			A	G	0.2	1.52	[1.35-1.72]
		GCST002299			A	G	0.19	1.64	NR
		GCST002073			A	G	0.19	1.62	NR
5	rs35923643	GCST004146	11	123484683	G	A	NR	1.63	[1.53-1.72]
		GCST003468			G	A	0.196	1.66	[1.54-1.79]
6	rs10849033	GCST000464	12	4315956	A	G	0.02	2.55	[1.60-3.80]
7	rs2267708	GCST004146	7	124752458	T	C	NR	1.16	[1.1-1.22]
8	rs7578199	GCST003468	2	241253433	C	T	0.745	1.176471	[1.11-1.25]
9	rs114961115	GCST90011814	6	46182333	A	G	0.027043	1.817851	NR
10	rs4266947	GCST007540	1	202233110	T	C	NR	4.62	[2.37-9.01]
11	rs267759	GCST000323	5	36137518	G	A	NR	3.23	[1.52-6.87]
12	rs630662	GCST001320	8	107960070	G	A	0.29	-	-
13	rs139996880	GCST003468	5	1284538	A	G	0.165	1.29	[1.18-1.42]
14	rs1359742	GCST003468	9	22336997	C	G	0.499	1.204819	[1.12-1.28]
15	rs7090445	GCST002158	10	61961417	T	C	NR	-	-
16	rs7818688	GCST002792	8	95011854	A	C	NR	4.26	[2.45-7.42]

Note: RA: risk allele. OA: other allele. RAF: risk allele frequency in controls. OR: odds ratio. CI: 95% confidence interval of OR.

Supplementary Table 5. Supplementary information of Table 5

No	rsID	Study accession	Chr	Pos	RA	OA	RAF	OR	CI
1	rs73195662	GCST007540	7	106018014	G	C	NR	3.52	[2.12-5.85]
2	rs73718779	GCST003468	6	2969044	T	C	0.11	1.26	[1.16-1.36]
3	rs4459895	GCST004099	3	188236626	C	A	NR	-	-
4	rs10949482	GCST007149	6	18121083	T	C	NR	-	-
		GCST007148			T	C	NR	-	-
5	rs187021028	GCST90011814	19	22616454	A	T	0.01356	2.570694	NR
6	rs4525246	GCST004099	11	123524538	C	G	NR	-	-
7	rs79050301	GCST007150	6	18151959	C	T	NR	-	-
8	rs1142345	GCST003609	6	18130687	C	T	NR	-	-
9	rs1036935	GCST004146	18	50317164	G	A	0.22	1.15	[1.10-1.21]
10	rs7578199	GCST003468	2	241253433	C	T	0.745	1.176471	[1.11-1.25]

Note: RA: risk allele. OA: other allele. RAF: risk allele frequency in controls. OR: odds ratio. CI: 95% confidence interval of OR.
